# Abnormal pulmonary function tests predict the development of radiation-induced pneumonitis in advanced non-small cell lung Cancer

**DOI:** 10.1186/s12931-018-0775-2

**Published:** 2018-04-24

**Authors:** L. Torre-Bouscoulet, W. R. Muñoz-Montaño, D. Martínez-Briseño, F. J. Lozano-Ruiz, R. Fernández-Plata, J. A. Beck-Magaña, C. García-Sancho, A. Guzmán-Barragán, E. Vergara, M. Blake-Cerda, L. Gochicoa-Rangel, F. Maldonado, M. Arroyo-Hernández, O. Arrieta

**Affiliations:** 1 Subdirección de Investigación Clínica, INER, Calz. de Tlalpan 4502, Tlalpan, Sección XVI, C.P. 14080, Ciudad de México, México; 2 Departamento de Fisiología Respiratoria INER, México city, México; 3 Unidad Funcional de Oncología Torácica, Instituto Nacional de Cancerología (INCAN), Av. San Fernando No. 22, Col. Sección XVI, Tlalpan, 14080 Ciudad de México, CP Mexico; 4 Departamento de Investigación en Epidemiología y Ciencias Sociales en Salud, Instituto Nacional de Enfermedades Respiratorias (INER), México city, Mexico; 5 Departamento de Radio-Oncología, INCAN, México city, Mexico; 6 Thoracic Oncology Unit, Instituto Nacional de Cancerología, Ciudad de México, México

**Keywords:** Pneumonitis, Radiotherapy, Non-small cell lung Cancer, Pulmonary function tests

## Abstract

**Background:**

Radiation pneumonitis (RP) is a frequent complication of concurrent chemoradiotherapy (CCRT) and is associated with severe symptoms that decrease quality of life and might result in pulmonary fibrosis or death. The aim of this study is to identify whether pulmonary function test (PFT) abnormalities may predict RP in non-small cell lung cancer (NSCLC) patients.

**Methods:**

A prospective multi-institutional study was conducted with locally advanced and oligometastatic NSCLC patients. All participants were evaluated at baseline, end of CCRT, week 6, 12, 24, and 48 post-CCRT. They completed forced spirometry with a bronchodilator, body plethysmography, impulse oscillometry, carbon monoxide diffusing capacity (DLCO), molar mass of CO_2_, six-minute walk test and exhaled fraction of nitric oxide (FeNO). Radiation pneumonitis was assessed with RTOG and CTCAE. The protocol was registered in www.clinicaltrials.gov (NCT01580579), registered April 19, 2012.

**Results:**

Fifty-two patients were enrolled; 37 completed one-year follow-up. RP ≥ Grade 2 was present in 11/37 (29%) for RTOG and 15/37 (40%) for CTCAE. Factors associated with RP were age over 60 years and hypofractionated dose. PFT abnormalities at baseline that correlated with the development of RP included lower forced expiratory volume in one second after bronchodilator (*p* = 0.02), DLCO (*p* = 0.02) and FeNO (*p* = 0.04). All PFT results decreased after CCRT and did not return to basal values at follow-up.

**Conclusions:**

FEV_1_, DLCO and FeNO prior to CCRT predict the development of RP in NSCLC. This study suggests that all patients under CCRT should be assessed by PFT to identify high-risk patients for close follow-up and early treatment.

**Electronic supplementary material:**

The online version of this article (10.1186/s12931-018-0775-2) contains supplementary material, which is available to authorized users.

## Background

Lung cancer is the main cause of cancer-related mortality worldwide [1]. Non-small cell lung cancer (NSCLC) represents approximately 75% of the histological types of lung cancer. Despite the development of new diagnostic tools, in México most cases are diagnosed in an advanced stage [2]. Concurrent chemoradiotherapy (CCRT) is the standard treatment for locally advanced NSCLC [3], but the advantage in survival seen with CCRT is contrasted with an increase in toxicity [4, 5]. Carboplatin with paclitaxel is one of the most commonly used schemes during CCRT, and it has demonstrated similar efficacy and less toxicity compared with etoposide and cisplatin when used with concurrent radiotherapy (RT) [6]. Furthermore, patients with oligometastatic disease can receive local control with CCRT, which might improve outcome [7–11].

Exposure to radiation frequently induces pulmonary toxicity, which can present as pneumonitis in a range from 15% and even up to 58% of the patients who receive radiotherapy, affecting quality of life (QoL) and oxygen dependence and leading to death in up to 50% of cases [12–14]. Several studies have identified patient-related factors and the association between dose of RT administered and the radiation pneumonitis (RP) rate; however, predictive models have not been widely applied, as some of the research available includes heterogeneous groups of patients, radiation monotherapy, or non-lung malignancies. Thus, the correlation between pretreatment variables and the development of pneumonitis is not clear [12, 15].

A meta-analysis described that age over 65 years, dosimetric lung volume receiving ≥20 Gy (V20) and CCRT schemes were predictive factors for RP [16]. Other studies did not find associations between age and RP [17–19]. V20 and mean lung dose (MLD) are the most consistent factors associated with RP in the literature [20]. Other factors associated with high RP frequency are related with gemcitabine [21].

Forced expiratory volume in one second (FEV_1_) and carbon monoxide diffusing capacity (DLCO) are considered the mainstays for patient selection before major lung resection, although patients who do not meet the expected values may be candidates for CCRT. Still, there is no consistent evidence that supports their association and the development of RP. Some investigations reported that pulmonary function tests (PFT) predict RP with lower baseline FEV_1_ [19, 22] and that FEV_1_% [23] and DLCO% [24, 25] were significantly associated with risk of RP. A recent study with 260 patients showed that lower FEV_1_ is associated with a lower risk of RP [26], while other studies did not show significant correlations between PFT and RP [12, 27, 28].

Multiple studies reported the incidence of RP, although data was limited due to the number of patients, incomplete assessment of pulmonary functions, several chemotherapy schemes, different radiation therapy techniques and retrospective studies [4, 16, 29]. Despite all the available data, no study was specifically designed to describe the relationship between the incidence of RP and prediction values for pulmonary function tests.

The objective of this study was to determine whether baseline pulmonary function tests could identify patients with a high risk of developing RP after CCRT with carboplatin and paclitaxel in NSCLC patients with locally advanced and oligometastatic disease. Likewise, we describe the performance of spirometry with a bronchodilator, body plethysmography, impulse oscillometry, carbon monoxide diffusion capacity (DLCO), molar mass of carbon dioxide (CO_2_), six-minute walk test and exhaled fraction of nitric oxide (FeNO).

## Methods

### Study design

A prospective, multi-institutional cohort study was conducted in patients with locally advanced NSCLC according to TNM 7 (clinical stage IIIA and IIIB) and oligometastatic disease (clinical stage IV) treated with CCRT from June 2013 to June 2015 at the Instituto Nacional de Cancerología in Mexico. Patients with oligometastatic disease were assessed by the multidisciplinary oncologic team to determine local control. The protocol was approved by the Scientific and Bioethical committees of the Instituto Nacional de Cancerología (013/014/ICI; CEI/799) and the Instituto Nacional de Enfermedades Respiratorias in Mexico (C12–12). The protocol is registered in www.clinicaltrials.gov (NCT01580579). All patients signed informed consent and then were evaluated six times, on the initial visit, at the end of CCRT and at 6, 12, 24 and 48 weeks post-CCRT. At each visit, pulmonary function tests (spirometry with bronchodilator test, body plethysmography, impulse oscillometry, DLCO, FeNO, molar mass of carbon dioxide and six-minute walk test) were performed. Lung toxicity was evaluated with the Common Terminology Criteria for Adverse Events (CTCAE) V.4.0 and Radiation Therapy Oncology Group (RTOG). In this work, RP was considered to be pneumonitis grade 2 to 5 according to both scales.

### Chemotherapy

Paclitaxel 50 mg/m2 was administered weekly for 6 weeks as an intravenous (IV) infusion over 1 h on days 1, 8, 15, 22, 29, and 36 of the planned radiation course. All patients received the following premedications 1 h before the paclitaxel infusion: dexamethasone 20 mg IV; diphenhydramine 25 mg IV; and ranitidine 50 mg IV. After the paclitaxel infusion, weekly carboplatin at AUC 2.0, was delivered as an IV bolus infusion over 30 min. Dose modifications were made in patients presenting grade 3 toxicity, restarting the treatment when the toxicity improved to grade 2.

### Radiotherapy

The patients went through 3D simulation, radiotherapy design and plan calculation was calculated with Varian Eclipse v.11.0 with dose corrected for tissue heterogeneity. Radiation therapy was administered 5 days per week (i.e., Monday to Friday) in 2 or 2,5Gy fractions daily by use of 6-18MV X rays, using Varian IX or C-2100 linear accelerators. Cone-beam CT was performed once a week. Three-dimensional conformal and intensity modulated radiation therapy were allowed. Radiation doses were prescribed to the planning target volume (PTV). The gross tumor volume was defined as the primary tumor and regionally involved nodes on CT when 1 cm or larger, or SUV uptake > 3. Clinical target volume margins were 0.5–1.0 cm, and PTV margins were 0.5–1.0 cm as well. The administered doses were 50 to 66 Gy in 20–33 fractions, and the following volume dose restrictions were recommended: 35% of affected lung parenchyma under 20 Gy (V20 < 35%) and 65% of lung parenchyma under 5 Gy (V5 < 65%) and a MLD of 20 Gy. Elective nodal irradiation was not permitted.

### Statistical analysis

For descriptive purposes, continuous variables are presented as arithmetic means and standard deviation (SD). Median and quartile values were used to compare the medians considered to assess the pulmonary functions in each visit. To assess the differences throughout the study period, the Friedman test was used. Fisher’s exact test was performed for categorical variables, and the Wilcoxon signed-rank test was used to analyze the changes in PFT continual variables. Cutoff points for PFTs were obtained using the Cutoff Finder version 2.1 [30] A value of *p* ≤ 0.05 was considered statistically significant. Statistical tests were performed using STATA software ver. 12 (StataCorp, Lakeway Drive College Station, Texas, USA).

## Results

### Patients

**Fig. 1:**
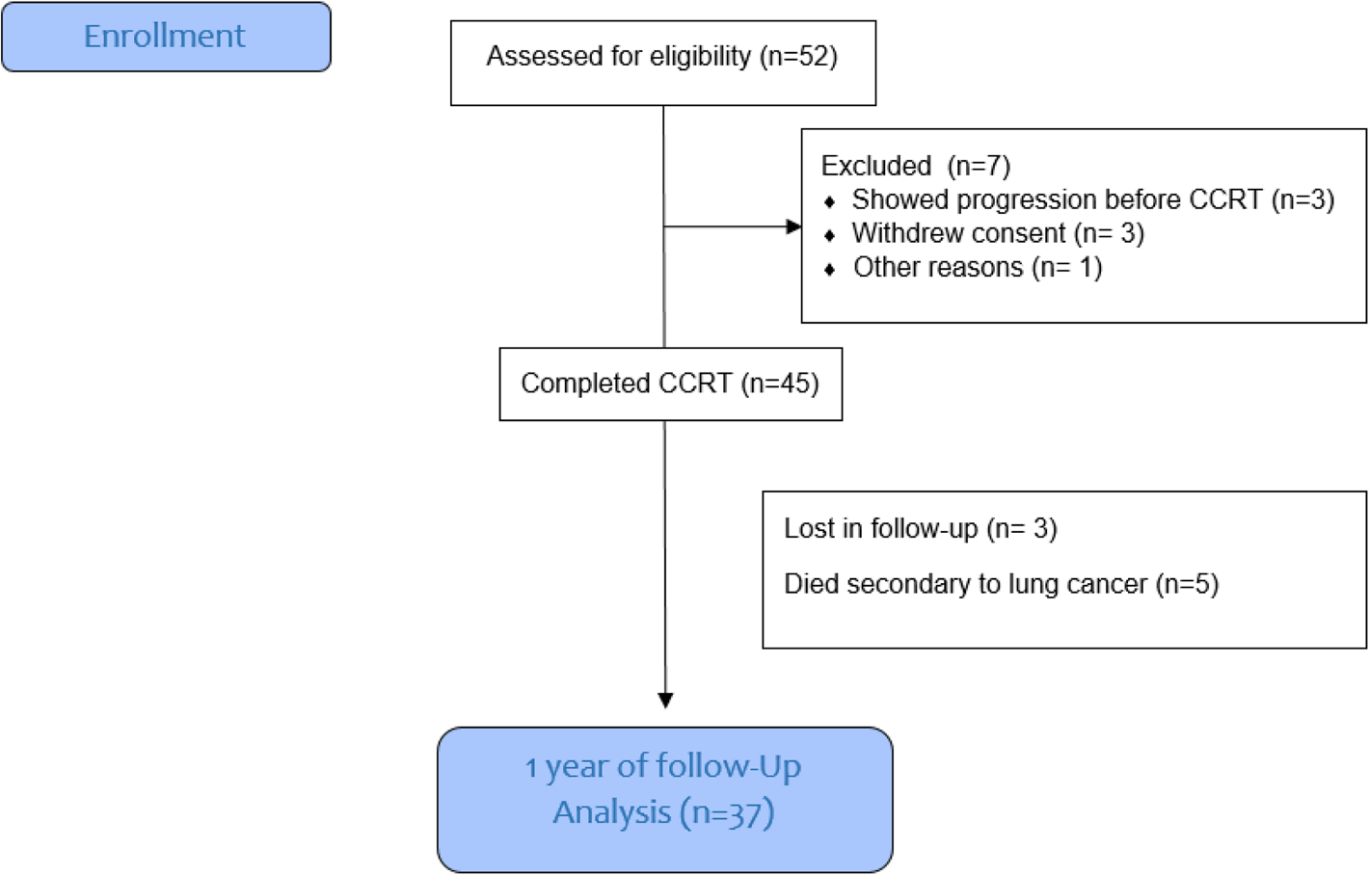
Consort flow diagram of the patients enrolled in this study

A total of 52 patients were enrolled in the study, and 37 completed a one-year follow-up (CONSORT chart Fig. 1). The characteristics of patients at baseline are shown in Table 1. There was male predominance (21 males (56.8%) vs. 16 females (43.2%) and the majority of patients were 60 years or older (64.9%). Most patients had a smoking history (*n* = 20, 54.1%) and a histological diagnosis of adenocarcinoma (*n* = 25, 67.6%). Seventeen (45.9%) patients presented locally advanced NSCLC and 20 patients (54.1%) presented oligometastatic disease. The most common tumor localization was reported in the upper lobes (15, 40.5%), followed by lower (13, 35.2%) and medium (9, 24.3%) lobes.

**Table 1:** Patient demographics and Tumor characteristics

Variable		Patients enrolled *n* = 52 (%)	Patients with one-year follow-up *n* = 37(%)
Age years	≤ 60 ≥ 60	20 (38.4) 32 (61.6)	13 (35.1) 24 (64.9)
Sex	Female Male	24 (45.3) 28 (53.8)	16 (43.2) 21 (56.8)
^a^ECOG	0–1 > 2	46 (88.5) 6 (11.5)	34 (91.9) 3 (8.1)
Smoking history	Yes No	22 (42.3) 30 (57.7)	20 (54.1) 17 (45.9)
Current smoking	Yes No	7 (13.5) 45 (86.5)	5 (13.5) 32 (86.5)
Tobacco index	Package/Year	38 (0.3–111)	34(0.3–111)
Histology	Adenocarcinoma Others	35 (67.3) 17 (32.7)	25 (67.6) 12 (32.4)
^b^Clinical stage	III IV	26 (50) 26 (50)	17 (45.9) 20 (54.1)
^c^Localization	Superior Medium Inferior	11 (21.1) 12 (23.1) 29 (55.8)	15 (40.5) 9 (24.3) 13 (35.2)

^a^ECOG (Eastern Cooperative Oncology Group), performance status 0-fully active, 1-Restricted in physically strenuous activity, 2-Ambulatory and capable of all selfcare, 3-Capable of only limited selfcare, 4-Completely disabled and 5-Dead. ^b^7th lung cancer TNM classification and staging system. ^c^CT scan tumor localization

### Radiation pneumonitis incidence

The incidence of RP varied according to the scale used. With the RTOG scale, 32/37 (86.4%) patients developed RP (Grade 1, 56.7%; Grade 2, 24.3%; Grade 3, 2.7%; Grade 4, 2.7%), while with the CTCAE scale, 32/37 (86.4%) patients developed RP (Grade 1, 45.9%; Grade 2, 32.4%; Grade 3, 5.4%; Grade 4, 2.7%). RP frequency with the RTOG scale was associated with patients over 60 years old (*p* = 0.03). With the CTCAE scale, the development of RP was associated with patients over 60 years (*p* = 0.02) (Additional file 1: Table S1).

Baseline dosimetry characteristics comparing patients with the development of RP (grade ≥ 2) are summarized in Table 2. The hypofractionated dose of 250 cGy was correlated with the development of RP according to the CTCAE scale (*p* = 0.036). With the RTOG scale, the development of RP was associated with PTV ≥350 cm^3^ (*p* = 0.013), V5 ≥ 65%, and V20 ≥ 35%, and MLD over 20 Gy were not associated with RP development in any scale (Table 3).

**Table 2:** Dosimetric baseline characteristics of patients who developed RP and non-RP patients

Variable	RTOG			CTCAE		
	No pneumonitis *n* = 26	Pneumonitis *n* = 11	*P* value	No pneumonitis *n* = 22	Pneumonitis *n* = 15	*P* value
GTV (cm^3^)	75.6 (41.3134)	135 (49.4265)	0.17	80.8 (39.8135)	112 (41.4252)	0.52
PTV (cm^3^)	321 (216,420)	444 (267,581)	0.14	322 (221,428)	392 (194,581)	0.3
Absolute dose (Gy)						
≤ 60	7 (26.9)	5 (45.5)	0.43	5 (22.7%)	7 (46.7%)	0.12
> 60	19 (73.1)	6 (54.5)		17 (77.3%)	8 (53.3%)	
Dose per fraction (cGy)						
180–200	20 (76.9%)	5 (45%)	0.12	18 (81.8%)	7 (50%)	**0.036**
250	6 (23.1%)	6 (54%)		4 (18.2%)	8 (50%)	
Restriction dose (%)						
V 5	65 (56.5,77.9)	54.9 (47, 69.4)	0.17	65.5 (56,79)	56.8 (48,70)	0.19
V 20	49.7 (41, 57.2)	42 (27.7,55.5)	0.4	49.7 (39,57.9)	44 (32,59.4)	0.62
MLD (Gy)	26 (18.5, 29.2)	20 (13,30)	0.57	25.8 (18,29.6)	21.4 (15,29)	0.53

*GTV* Gross tumoral volume, *PTV* Planning tumor volume, *V 5* Volume of lung receiving at least 5 Gy, *V 20* Volume of lung receiving at least 20 Gy, *MLD* Mean lung dose

Significant *P* values in bold

**Table 3:** Dosimetric baseline characteristics of patients who developed RP

Variable		RTOG			CTCAE		
		No RP *n* = 26 (%)	Pneumonitis *n* = 11 (%)	*P* value	No RP *n* = 22 (%)	Pneumonitis *n* = 15 (%)	*P* value
GTV (cm^3^)	0–99.9	16 (61.5)	4 (36.4)	0.078	13 (59.1)	6 (40)	0.324
	≥100	10 (38.5)	7 (63.6)		9 (40.9)	9 (60)	
PTV	0–349.9	19 (73.1)	4 (36.4)	**0.013**	16 (72.3)	6 (40)	0.086
	≥350	7 (26.9)	7 (63.6)		6 (27.3)	9 (60)	
V 5 (%)	0–64.9	13 (50)	8 (72.7)	0.284	11 (50)	10 (66.7)	0.50
	≥65	13 (50)	3 (27.3)		11 (50)	5 (33.3)	
V 20 (%)	0–34.9	4 (15.4)	3 (27.3)	0.648	4 (18.2)	3 (20)	0.999
	≥35	22 (84.6)	8 (72.7)		18 (81.8)	12 (80)	
MLD (Gy)	0–19.9	10 (38.5)	5 (45.5)	0.727	9 (40.1)	7 (46.7)	0.748
	≥20	16 (61.5)	6 (54.5)		13 (59.1)	8 (53.3)	

*GTV* Gross tumoral volume, *PTV* Planning tumor volume, *V 5* Volume of lung receiving at least 5 Gy *V 20* Volume of lung receiving at least 20 Gy, *MLD* Mean lung dose

Significant *P* values in bold

### Pulmonary function tests

**Fig. 2:**
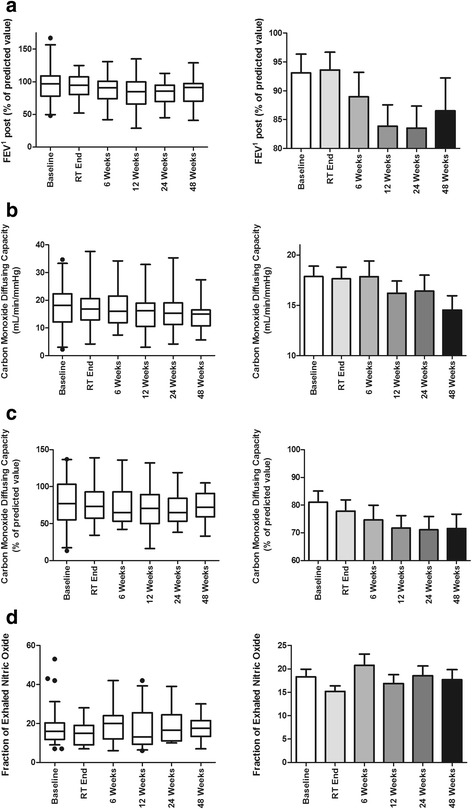
Changes in PFT tests during follow up, (**a**) FEV1, (**b**) Carbon Monoxide Diffusing Capacity, (**c**) Fraction of exhaled nitric oxide

The analysis of baseline characteristics between patients who developed RP and non-RP patients (Table 4) showed a correlation between baseline FEV_1_ in post-bronchodilator spirometry (% of predicted value) and FeNO with the development of RP using both RTOG (*p* = 0.02 and *p* = 0.01, respectively) and CTCAE scales (*p* = 0.02 and *p* = 0.04, respectively). The ratio of the FEV_1_ in post-bronchodilator spirometry (% of predicted value) and FVC had statistical significance using the RTOG scale (*p* = 0.01). Lower values of DLCO (mL/min/mmHg) and DLCO (% predicted) in the baseline test correlated with RP only in the CTCAE scale (p = 0.02 and 0.049, respectively). According to the RTOG scale, the *p* value was borderline (*p* = 0.06 and 0.06, respectively). The best cutoff point values associated with RP development were FEV_1_ ≤ 1.9 (Lts.) for the RTOG scale (HR 1.35, 95% CI 0.72–2.51), FEV_1_ ≤ 1.9 (Lts) for the CTCAE scale (HR 3.21, 95% CI 0.93–11.16); FeNO ≥18.5 for the RTOG scale (HR 1.99, 95% CI 1.22–3.24), FeNO ≥17.5 for the CTCAE scale (HR 1.9, 95% CI 1.10–3.28), DLCO ≤16.9 for the RTOG scale (HR 1.81, 95% CI 0.97–3.34), and DLCO ≤18.9 for CTCAE scale (HR 2.26, 95% CI 1.21–4.22). These data are described in Table 5. Other tests, such as plethysmography, molar mass of CO_2_, impulse oscillometry, and the 6-min walk test did not have any association with patients at high risk for RP. Figure 2 shows the changes after CCRT in FEV_1_, FeNO and DLCO.

**Table 4:** Comparison of baseline pulmonary function tests results using RTOG and CTCAE scales

	RTOG Scale*			CTCAE Scale*		
Variable	No pneumonitis (*n* = 26)	Pneumonitis (*n* = 11)	*P* value	No pneumonitis (*n* = 22)	Pneumonitis (*n* = 15)	*P* value
	median (p25, p75)	median (p25, p75)		median (p25, p75)	median (p25, p75)	
Spirometry						
FEV1 post (% predicted value)	98 (84, 110)	73 (60, 92)	**0.02**	98 (86, 110)	83 (61, 100)	**0.02**
FVC post (% predicted value)	106 (90, 114)	91 (79, 114)	0.17	109 (90, 116)	93 (79, 105)	0.06
FEV1/FVC post (%)	77 (72, 81)	65 (54, 74)	**0.01**	75 (70, 82)	70 (58, 79)	0.07
Plethysmography						
TLC (L)	5.1 (4.4, 6.3)	6.8 (4.8, 6.9)	0.06	5.5 (4.7, 6.3)	5.5 (4, 6.9)	0.93
TLC (% predicted value)	101 (88, 111)	102 (94, 113)	0.63	103 (95, 113)	99 (86, 111)	0.25
RV/TLC (%)	39 (35, 44)	50 (38, 59)	0.01	38 (34, 44)	45 (38, 56)	0.02
Impulse oscillometry						
Rrs5 (Kpa/L/s)	0.34 (0.29, 0.41)	0.33 (0.28, 0.44)	0.98	0.34 (0.29, 0.42)	0.33 (0.28, 0.4)	0.63
Rrs20 (Kpa/L/s)	0.26 (0.22, 0.31)	0.27 (0.2, 0.31)	0.71	0.27 (0.22, 0.32)	0.26 (0.21, 0.29)	0.47
Xrs5 (Kpa/L/s)	−0.13 (−0.17, −0.1)	−0.13 (−0.17, −0.082)	0.64	−0.13 (−0.17, −0.1)	−0.13 (−0.17, −0.09)	0.67
AX (Kpa/L)	0.55 (0.45, 1.3)	0.81 (0.4, 1.2)	0.88	0.55 (0.45, 1.3)	0.67 (0.47, 1.2)	0.77
Carbon Monoxide Diffusing Capacity						
DLCO (mL/min/mmHg)	20 (17, 24)	16 (10, 19)	0.06	22 (18, 25)	16 (12, 19)	**0.02**
DLCO (% predicted value)	91 (76, 110)	66 (51, 98)	0.06	92 (76, 111)	71 (58, 98)	**0.049**
Molar mass of carbon dioxide						
PO2 (mmHg)	66 (61, 69)	60 (52, 68)	0.14	67 (61, 70)	60 (57, 68)	0.07
pCO2 (mmHg)	29 (27, 31)	31 (26, 34)	0.17	30 (25, 32)	29 (27, 33)	0.65
SO2 (%)	92 (91, 94)	92 (87, 94)	0.32	92 (91, 94)	92 (87, 94)	0.21
Six-minute walk						
Distance (m)	487 (379, 510)	429 (367, 481)	0.16	495 (369, 510)	435 (404, 482)	0.34
Fraction of exhaled nitric oxide						
FENO	15 (10, 18)	24 (19, 53)	**0.01**	15 (11, 18)	23 (16, 30)	**0.04**

*FEV* Forced expiratory volume, *FVC* Forced vital capacity, *FEV*_*1*_, forced expiratory volume in one second, *TLC* Total lung capacity, *RV* Residual volume, *Rrs5* Resistance at 5 Hz, *Rrs20* Resistance at 20 Hz, *Xrs5* Reactance at 5 Hz, *AX* Reactance Area, *DLCO* Diffusing capacity of the lung for CO, *FeNO* Fraction of exhaled Nitric Oxide. * Wilcoxon rank-sum test for unmatched data was used to compare respiratory variables

Significant *P* values in bold

**Table 5:** Univariable analysis for cutoff points of Pulmonary Function tests and Pneumonitis

RTOG Scale						
PFT	Cutoff point	No Pneumonitis	Pneumonitis	HR	95% CI	*P* value
FEV1	˂1.9	5 (19.2%)	4(36.4%)	1.35	0.72–2.51	0.404
	≥1.9	21 (80.8%)	7 (63.6%)			
FeNO	˂18.5	17 (65.4%)	1 (9.1%)	1.99	1.22–3.24	**0.003**
	≥18.5	9 (34.6%)	10 (90.9%)			
DLCO	˂16.9	6 (23.1%)	7 (63.6%)	1.81	0.97–3.34	**0.028**
	≥16.9	20 (76.9%)	4 (36.4%)			
CTCAE Scale						
PFT	Cutoff point	No Pneumonitis	Pneumonitis	HR	95% CI	*P* value
FEV1	˂1.9	2 (9.1%)	7 (46.7%)	3.21	0.93–11.16	**0.017**
	≥1.9	20 (90.9%)	8 (53.3%)			
FeNO	˂17.5	13 (59.1%)	3 (20%)	1.90	1.10–3.28	**0.041**
	≥17.5	9 (40.9%)	12 (80%)			
DLCO	˂18.9	7 (31.2%)	12 (80%)	2.26	1.21–4.22	**0.007**
	≥18.9	15 (68.2%)	3 (20%)			

*FEV*_*1*_ forced expiratory volume in one second, *FeNO* Fraction of exhaled Nitric Oxide, *DLCO* Diffusing capacity of the lung for CO, *HR* Hazard ratio, *CI* confidence interval

Significant *P* values in bold

Overall PFT had detrimental changes with statistical significance, and none of them recovered to their baseline values. Patients who developed RP, or not, also presented decrease in the spirometry, plethysmography, and diffusion capacity of the lung for carbon monoxide using both scales. These changes were not associated with the development of RP grade ≥ 2 (Additional file 1: Table S1).

## Discussion

Radiation pneumonitis (RP) is the most important complication related to CCRT, as it impairs respiratory function and decreases the quality of life [13, 14, 31]. The incidence of RP fluctuates from 15 to 58%, and this range could be explained by its evaluation methods, symptom assessment, awareness of the disease and different radiation techniques [16, 31–33]. In this work, we found an incidence of 86.4% using the ROTG scale and 86.4% according to the CTCAE scale; however, RP with clinical relevance (Grade ≥ 2), was 29.7% for RTOG and 40.5% for CTCAE scales.

In our study, being 60 years of age or older was a predicting factor of RP, which is consistent with several publications that have documented age as a risk factor in radiotherapy [14, 16]; however, neither sex nor functional class were able to predict this complication. The association between smoking and radiation-induced lung toxicity was controversial, it has been reported that smoking history or active smoking are protective factors against pneumonitis [34]. This could be because smoking-damaged lungs may not be as sensitive to radiation injury as healthy lungs, and this may possibly be due to tobacco-induced immunosuppression or by the presence of nonfunctional airspace in these lungs [35, 36]. However, this study did not show any trend related to these factors. The frequency of smoking habit in our sample was low (52%), and this finding could be explained by other factors [37, 38].

Several studies have reported that CCRT affects the pulmonary function, but there is limited information on the adequate identification of high-risk patients who could develop RP [16]. With respect to the dosimetric variables of radiotherapy, we found that PTV greater than 350 is associated with RP; in this work, we did not find that non-compliance with recommended restriction doses (PA V5 < 65%, PAV20 < 35% and MLD < 20 Gy) were associated with the development of RP, although it has been previously reported [13, 16, 39]. Dose per fraction over 250 (cGy) was the only dosimetric variable that showed a statistically significant association with the development of RP, according to the CTCAE scale, this finding can be explained by the fact that hypofractionated doses have a great biological impact [40, 41]. Therefore, we suggest discontinuing this fractionation scheme, even in patients with palliative schemes.

Chronic obstructive pulmonary disease and pulmonary emphysema are common comorbidities in lung cancer [2], although many cancer patients do not have the criteria for these diseases, and alterations in PFT results are common. The baseline results of two of the PFTs showed that the spirometry and fractional concentrations of exhaled nitric oxide (FeNO) showed statistically significant differences between the patients who developed RP and the patients who did not develop RP, according to both scales; this implies that patients with impaired lung capacity prior to CCRT had a higher risk of presenting RP [42].

In the results of spirometry, alterations in FEV_1_ showed a statistically significant association with the development of PR; the higher this value is in the initial evaluation, the better the prognosis for the patients. One of the most interesting findings of this work is the relationship between FeNO and the development of PR, given that nitric oxide in expired air is a noninvasive marker of airway inflammation. It has been used in the diagnosis and follow-up of patients with inflammatory pulmonary diseases [43, 44]. Patients with elevated FeNO levels had an increased risk of developing RP according to both scales, which strongly suggests that those patients who already had a basal inflammatory reaction, such as emphysema or COPD, likely due to neoplasms or comorbidity, were more likely to develop PR. During follow-up, FeNO continued to increase, even exceeding 20% at its peak at week 6 of CCRT, given that inflammation was expected to decline after CCRT. According to anticipations, FeNO levels had a partial recovery but failed to reach the initial value. To the best of our knowledge, this is the first work that reports the relationship between FENO and RP development after CCRT. However, further studies are required to confirm this finding and support the addition of this test to the standard PFT battery [45, 46]. The results of the diffusion capacity of the lung for the carbon monoxide test (DLCO) showed that the patients who had a lower pulmonary diffusion of basal carbon monoxide and percentage of the predicted value developed RP using the CTCAE scale and was borderline with the RTOG scale. In a similar way, there are reports that suggest that impaired baseline DLCO could predict the development of RP, in the same way, it has been reported that small affectations in DLCO after SABR, can significantly impact the symptomatology of patients with NSCLC, which reinforces the idea of including this test in the close monitoring of patients with NSCLC [20, 25].

Our study shows that CCRT causes a decrease in the overall PFT results. For example, FEV_1_ was decreased to 12%, plethysmography (TLC) to 25% and diffusion capacity for carbon monoxide to 17%. In a previously compromised patient, small changes such as these can significantly affect the outcome and favor the presentation of important clinical changes and oxygen dependence. These changes occur in all patients, with or without RP, so that changes in PFT after CCRT were not associated with the development of RP.

The limitation of our study is its small sample size. Nevertheless, the evaluation of pulmonary function was comprehensive, and all the patients had a follow-up of at least one year.

## Conclusion

Age over 60 years and poor results in FEV_1_, DLCO and FeNO prior to CCRT predicted the development of RP in patients with NSCLC. This study suggests that all patients with advanced NSCLC who receive CCRT must be assessed by PFT before the start of treatment in order to identify patients at high risk for RP, provide a close follow-up, and consider the use of early treatment to reduce long-term complications. There is potential to investigate the utility of PFT as a predictor of pneumonitis. However, additional studies are required to accurately identify patients at greater risk and to provide effective preventive treatment.

## Additional file

Additional file 1: **Table S1**. Patient characteristics based on RTOG and CTCAE criteria. (DOCX 19 kb)

## Abbreviations

CCRT: Concurrent chemoradiotherapy
CO_2_: Carbon dioxide
CTCAE: Common Terminology Criteria for Adverse Events
DLCO: Carbon monoxide diffusion capacity
FeNO: Exhaled fraction of nitric oxide
FEV_1_: Forced expiratory volume in one second
MLD: Mean lung dose
NSCLC: Non-smalls Cell Lung Cancer
PFT: Pulmonary function tests
QoL: Quality of life
RP: Radiation pneumonitis
RT: Radiotherapy
RTOG: Radiation Therapy Oncology Group
