# Supplementary material for: Abnormal pulmonary function tests predict the development of radiation-induced pneumonitis in advanced non-small cell lung Cancer
Source: Respir Res. 2018 Apr 24;19:72. doi: 10.1186/s12931-018-0775-2 (PMC5937833; doi:10.1186/s12931-018-0775-2)
Supplement: Supplementary file 1 — Table S1. Patient characteristics based on RTOG and CTCAE criteria. (DOCX 19 kb) [file 12931_2018_775_MOESM1_ESM.docx]

Additional file 1: Table S1 Patient characteristics based on RTOG and CTCAE criteria

| Variable | RTOG | | | CTCAE | | |
| --- | --- | --- | --- | --- | --- | --- |
|  | No pneumonitis n=26 (%) | Pneumonitis n=11 (%) | P value | No pneumonitis n=22 (%) | Pneumonitis n=15 (%) | P value |
| **Age** | | | | | | |
| ≤ 60 | 12 (46.2) | 1 (9.1) | **0.03** | 11 (50) | 2 (13.3) | **0.02** |
| >60 | 14 (53.8) | 10 (90.9) |  | 11 (50) | 13 (86.7) |  |
| **Sex** | | | | | | |
| Female | 14 (53.8) | 3 (27.3) | 0.13 | 11 (50) | 6 (40) | 0.4 |
| Male | 12 (46.2) | 8 (72.7) |  | 11 (50) | 9 (60) |  |
| **ECOG** | | | | | | |
| 0-1 | 24 (92.3) | 10 (90.9) | 0.66 | 20 (90.9) | 14 (93.3) | 0.99 |
| >2 | 2 (7.7) | 1 (9.1) |  | 2 (9.1) | 1 (6.7) |  |
| **Smoking history** | | | | | | |
| Yes | 11 (42.3) | 5 (45.5) | 0.57 | 13 (59.1) | 9 (60) | 0.99 |
| No | 15 (57.7) | 6 (54.5) |  | 9 (40.9) | 6 (40) |  |
| **Current smoker** | | | | | | |
| Yes | 2 (7.7) | 3 (27.3) | 0.14 | 20 (90.9%) | 12 (80) | 0.38 |
| No | 24 (92.3) | 8 (72.7) |  | 2 (9.1%) | 3 (20) |  |
| **Tobacco Index** | | | | | | |
| N (Package/Year) | 30 (20- 50) | 35  (11.5-57.5) | 0.97 | 36 (20-60) | 34 (20-55) | 0.83 |
| **Histology** | | | | | | |
| Adenocarcinoma | 18 (69.2) | 7 (63.6) | 0.51 | 15 (68.2) | 10 (66.7) | 0.99 |
| Others | 8 (30.8) | 4 (36.4) |  | 7 (31.8) | 5 (33.3) |  |

| Variable | RTOG | | | CTCAE | | |
| --- | --- | --- | --- | --- | --- | --- |
|  | No pneumonitis n=26 (%) | Pneumonitis n=11 (%) | P value | No pneumonitis n=22 (%) | Pneumonitis n=15 (%) | P value |
| **Clinical stage** | | | | | | |
| III | 11 (42.3) | 6 (54.5) | 0.37 | 9 (40.9) | 8 (53.3) | 0.52 |
| IV | 15 (57.7) | 5 (45.5) |  | 13 (59.1) | 7 (46.7) |  |
| **Affected Lung*** | | | | | | |
| Right | 19 (73.1) | 6 (60) | 0.35 | 16 (72.7) | 9 (64.3) | 0.43 |
| Left | 7 (26.9) | 4 (40) |  | 6 (27.7) | 5 (35.7) |  |
| **Localization** | | | | | | |
| Lower lobe  Medium lobe | 16 (71.5) | 6 (54.5) | 0.56 | 13 (59.1) | 8 (53.3) | 0.92 |
| Upper lobe | 10 (38.5) | 5 (45.5) |  | 9 (40.9) | 7 (46.7) |  |
| **Location*** | | | | | | |
| Peripheral | 9 (34.6) | 3 (30) | 0.56 | 15 (68.2) | 9 (64.3) | 0.54 |
| Central | 17 (65.4) | 7 (70) |  | 7 (31.8) | 5 (35.7) |  |
| **Lymph nodes** | | | | | | |
| N0-N1 | 10 (38.5) | 4 (36.4) | 0.48 | 8 (36.3) | 6 (40) | 0.58 |
| N2-N3 | 16 (61.5) | 7 (63.6) |  | 14 (63.7) | 9 (60) |  |
| **Hemoglobin** | | | | | | |
| ≤ 12 | 8 (30.8) | 4 (36.4) | 0.51 | 7 (31.8) | 5 (33.3) | 0.99 |
| >12 | 18 (69.2) | 7 (63.6) |  | 15 (68.2) | 10 (66.7) |  |
| **Albumin** | | | | | | |
| ≤ 3.5 | 6 (23.1) | 4 (36.4) | 0.44 | 5 (22.7) | 5 (33.3) | 0.71 |
| >3.5 | 20 (76.9) | 7 (63.6) |  | 17 (77.3) | 10(66.7) |  |
| **BMI** | | | | | | |
| ≤ 25 | 11 (42.3) | 5 (45.5) | 0.99 | 11 (50) | 5 (33.3) | 0.5 |
| >25 | 15 (57.7) | 6 (54.5) |  | 11 (50) | 10 (66.7) |  |

* The data of a patient could not be recovered from the clinical file due to problems in the clinical file.
